# Elevated D-dimer levels in high-risk multiple myeloma patients: a systematic review and meta-analysis

**DOI:** 10.3389/fonc.2026.1899079

**Published:** 2026-09-09

**Authors:** Changming Sun, Yiqun Guo, Weichuan Zhao

**Affiliations:** Department of Laboratory Medicine, Affiliated Hospital of Chengde Medical University, Chengde, Hebei, China

**Keywords:** biomarker, D-dimer, disease severity, meta-analysis, multiple myeloma, thrombosis, venous thromboembolism

## Abstract

**Background:**

Patients with multiple myeloma (MM) exhibit elevated thrombotic risk and variable disease outcomes. D-dimer, a fibrin degradation product, may serve as a biomarker reflecting both hypercoagulability and disease severity. This systematic review and meta-analysis compared D-dimer levels between MM patient subgroups defined by either poor prognostic features (e.g., advanced ISS stage) or thrombotic events and reference groups, with detailed discussion of how D-dimer integration reshapes routine thromboprophylaxis strategies and upgrades classic prognostic staging tools (ISS, R-ISS, IMPEDE-VTE).

**Methods:**

We systematically searched PubMed, Embase, Web of Science, and Cochrane Library from 2010 to 2025 for studies comparing plasma D-dimer levels between high-risk MM patients, defined by advanced ISS stage, occurrence of venous thromboembolism (VTE), or as a predictor of mortality, and reference groups. The primary outcome was the standardized mean difference (SMD) in D-dimer levels. Random-effects meta-analysis was performed using the inverse-variance method. Subgroup analyses examined differences by sample type and assay methodology. Risk of bias was assessed using domains adapted from ROBINS-I.

**Results:**

Six studies comprising 697 patients (171 high-risk, 526 reference) met inclusion criteria. High-risk MM patients demonstrated significantly elevated D-dimer levels compared to reference groups (SMD 1.60, 95% CI 0.72–2.48, p<0.0001, I²=88.2%). The effect remained consistent across subgroup analyses by sample type (all plasma: SMD 1.60, 95% CI 0.72–2.48) and assay method (immunoturbidimetric: SMD 1.59, 95% CI 0.44–2.75; fluorescent immunoassay: SMD 1.64, 95% CI 0.78–2.49). Sensitivity analysis confirmed robustness of findings. Funnel plot asymmetry was minimal, although formal tests were not conducted given the limited number of studies.

**Conclusions:**

MM patients with poor prognostic features or who experience VTE exhibit substantially elevated D-dimer levels compared to lower-risk populations (SMD >1.5). Beyond confirming statistical significance, our results deliver actionable clinical insights: D-dimer supplementation optimizes risk stratification workflows built on ISS/R-ISS and refines personalized thromboprophylaxis regimens derived from the IMPEDE-VTE scoring model. D-dimer may serve as a useful biomarker for risk stratification in MM, though prospective validation studies are needed.

## 1 Introduction

Multiple myeloma (MM) represents a malignant proliferation of plasma cells accounting for approximately 10% of hematologic malignancies, with global incidence rates of 6–7 per 100,000 person-years (1, 2). Beyond the direct consequences of uncontrolled plasma cell growth including bone destruction, anemia, and renal impairment, MM patients face substantially elevated thrombotic risk, with venous thromboembolism (VTE) incidence rates ranging from 5% to 20%, depending on treatment regimens and patient characteristics (3, 4). This hypercoagulable state stems from multiple factors including tumor-associated procoagulant activity, treatment-related thrombogenicity (particularly with immunomodulatory drugs), immobility from skeletal complications, and systemic inflammation (5, 6).

D-dimer, a fibrin degradation product generated during fibrinolysis, serves as an established biomarker of activated coagulation and fibrin turnover (7). In cancer populations, elevated D-dimer levels have been associated with increased VTE risk, disease progression, and mortality (8, 9). Multiple studies have investigated D-dimer’s prognostic value in MM, yielding promising but heterogeneous results regarding its association with thrombotic events, disease stage, and clinical outcomes (10, 11). Accumulating evidence from both hematological and solid tumor studies has underscored D-dimer’s broader prognostic relevance, demonstrating that elevated levels correlate not only with thrombotic complications but also with overall disease aggressiveness and survival outcomes (12, 13). Recent investigations have demonstrated that incorporating D-dimer into clinical risk prediction models, such as the IMPEDE VTE score, significantly improves identification of MM patients at highest VTE risk, enabling more targeted thromboprophylaxis strategies (14, 15).

The International Staging System (ISS) and its 2015 revision (R-ISS) represent the current standard for MM prognostication, stratifying patients based on serum beta-2 microglobulin, albumin, lactate dehydrogenase, and high-risk cytogenetic abnormalities (16, 17). However, these systems do not explicitly incorporate coagulation biomarkers, despite accumulating evidence that thrombotic complications independently predict mortality in MM. Several recent studies have examined D-dimer levels across different MM patient subgroups defined by ISS stage, VTE occurrence, or survival status (10, 11, 18–23), but systematic synthesis of these data remains lacking. Of note, D-dimer may be markedly elevated in patients experiencing acute thrombotic events but only modestly increased in patients with advanced-stage disease or poor survival, reflecting distinct pathophysiological mechanisms: acute coagulation cascade activation in the former versus chronic inflammation and tumor burden–driven hypercoagulability in the latter. Therefore, distinguishing between D-dimer’s role as a predictor of thrombotic events versus a marker of tumor burden and disease progression is critical for its appropriate clinical application.

Understanding the magnitude of D-dimer elevation in high-risk versus lower-risk MM populations has important clinical implications. First, it could validate D-dimer as a biomarker reflecting disease severity beyond traditional staging systems. Specifically, pairing D-dimer with ISS/R-ISS fills a critical gap in conventional staging: ISS and R-ISS only reflect tumor burden and cytogenetic risk, while D-dimer adds a real-time readout of systemic hypercoagulability, a fatal adverse outcome driver independent of tumor stage. Second, quantifying the effect size would inform clinicians whether D-dimer measurements add meaningful discriminatory value for risk stratification. Clinically, this added discriminatory power directly modifies two core pillars of MM management: long-term prognostic judgment via staging systems and short-term VTE prevention via thromboprophylaxis planning. Third, establishing whether D-dimer differences remain consistent across various analytical platforms and sample types would support standardization efforts. Finally, demonstrating robust associations between D-dimer levels and high-risk disease features could justify prospective studies evaluating D-dimer-guided thromboprophylaxis strategies. For the widely used IMPEDE-VTE score, which relies on static clinical factors (age, renal function, immunomodulatory drug use, etc.), D-dimer acts as a dynamic, real-time modifier to upgrade risk tiers and adjust anticoagulant intensity.

This systematic review and meta-analysis aimed to synthesize available evidence comparing D-dimer levels between high-risk and reference MM patient groups, where high-risk status was defined by poor prognostic features (e.g., ISS stage III), thrombotic events, or as a predictor of mortality, quantify the magnitude of difference using standardized mean difference estimates, explore sources of heterogeneity through subgroup analyses, and assess the quality of current evidence to guide future research directions, and elaborate on how statistically significant D-dimer disparities translate to tangible adjustments in standard MM clinical workflows, including optimization of established prognostic staging frameworks and personalized thromboprophylaxis protocols.

## 2 Methods

### 2.1 Protocol and registration

This systematic review and meta-analysis was conducted according to the Preferred Reporting Items for Systematic Reviews and Meta-Analyses (PRISMA) 2020 guidelines. A protocol was prepared *a priori* but was not prospectively registered in PROSPERO or any other registry. The absence of prospective registration is acknowledged as a methodological limitation, as such registration enhances transparency and reduces the potential for reporting bias.

### 2.2 Literature search strategy

**Figure 1:**
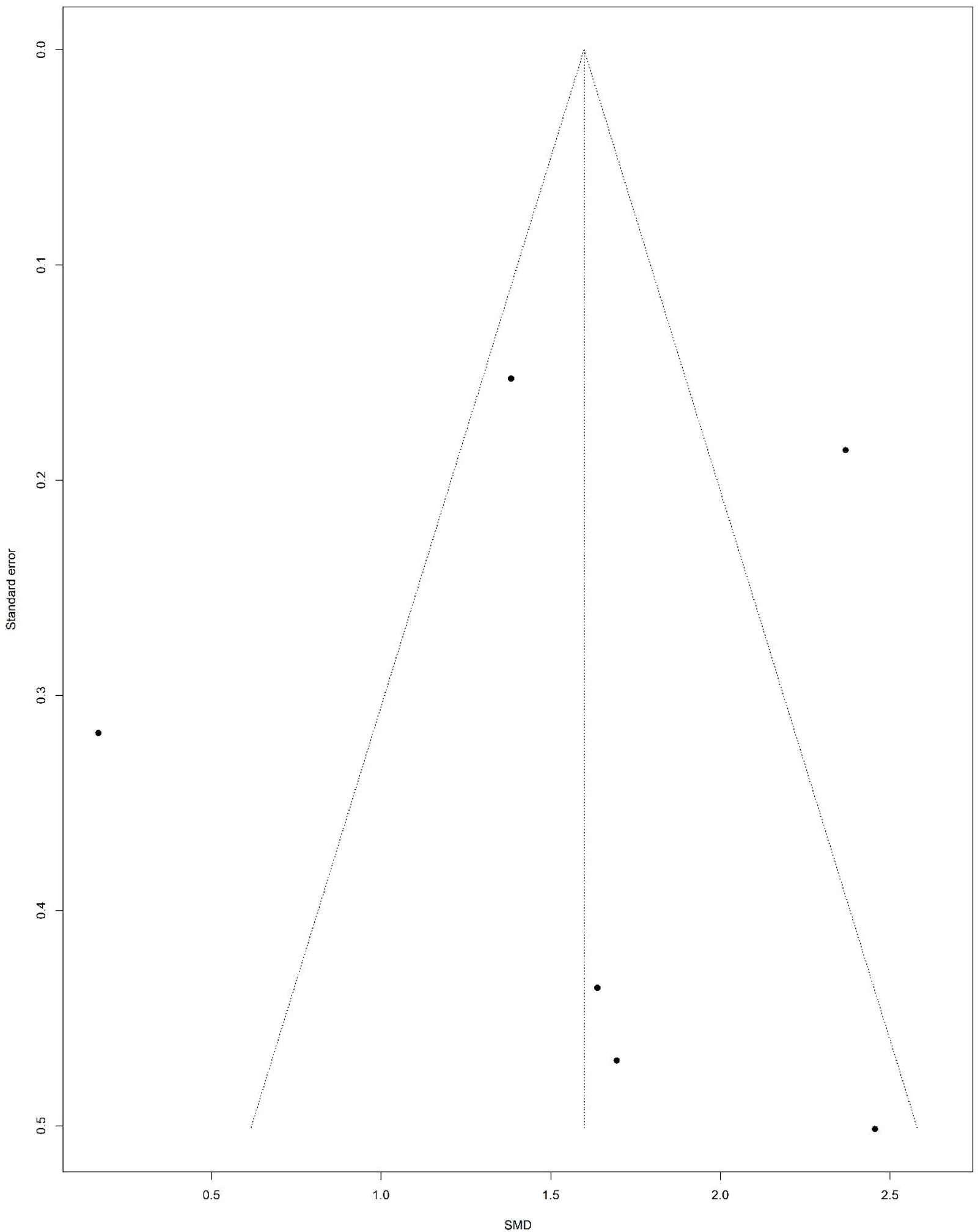
Funnel plot for D-dimer SMD.

We systematically searched PubMed/MEDLINE, Embase, Web of Science Core Collection, and Cochrane Library from January 1, 2010 to March 1, 2025. These four databases were selected to ensure comprehensive coverage: PubMed/MEDLINE provides broad biomedical literature indexing, Embase supplements with stronger pharmacological and European journal coverage, Web of Science captures high-impact multidisciplinary research, and the Cochrane Library identifies systematic reviews and clinical trials relevant to clinical practice. The 2010 start date was selected because modern MM treatment paradigms incorporating novel agents (immunomodulatory drugs, proteasome inhibitors) became standard after this time, and earlier cohorts would not reflect contemporary practice patterns. To reduce publication bias, we additionally screened ClinicalTrials.gov for ongoing or unpublished trials and manually searched reference lists of relevant reviews and included studies. One study (Hussein 2020 (20)) was identified through citation searching of reference lists rather than through the primary database searches, and this is reflected in the PRISMA flow diagram (**Figure 1**).

The search strategy combined Medical Subject Headings (MeSH) terms and free-text keywords. For PubMed, the following strategy was used: (“multiple myeloma”[MeSH] OR “multiple myeloma”[tiab] OR “plasma cell myeloma”[tiab] OR “myelomatosis”[tiab]) AND (“D-dimer”[tiab] OR “D dimer”[tiab] OR “fibrin degradation products”[MeSH] OR “FDP”[tiab]) AND (“venous thromboembolism”[MeSH] OR “VTE”[tiab] OR “deep vein thrombosis”[tiab] OR “DVT”[tiab] OR “pulmonary embolism”[tiab] OR “thrombosis”[MeSH] OR “coagulation”[tiab] OR “hypercoagul*”[tiab] OR “ISS staging”[tiab] OR “International Staging System”[tiab] OR “disease severity”[tiab] OR “prognosis”[MeSH] OR “mortality”[MeSH] OR “survival”[tiab]). Search strategies were adapted to each database’s syntax. The search was restricted to human studies but no language restrictions were initially applied at the search stage. Conference abstracts, editorials, and letters were screened only to identify additional full-text articles through reference tracking.

### 2.3 Eligibility criteria

Studies were considered eligible if they met all of the following criteria using a Population, Exposure, Comparator, Outcome, and Study design (PECOS) framework: (1) Population: Adult patients aged 18 years or older with diagnosed MM according to International Myeloma Working Group criteria; (2) Exposure: Plasma or serum D-dimer measured using any validated laboratory assay (immunoturbidimetric, latex agglutination, fluorescent immunoassay, or ELISA) at baseline or during treatment; (3) Comparator: Studies must include both a high-risk group and a reference (lower-risk) group. High-risk was defined as (a) advanced ISS stage (ISS III), (b) occurrence of VTE, or (c) patients who died (with mortality as the outcome being predicted by D-dimer). We acknowledge that these definitions capture distinct biological phenomena—tumor burden-driven hypercoagulability versus acute thrombotic events versus systemic disease aggression—and this inherent heterogeneity is a major focus of our analysis. Given the limited published literature in this emerging field, pooling these different high-risk definitions under a single composite category was a necessary methodological compromise to generate preliminary pooled estimates. To address this heterogeneity, we prespecified extensive subgroup analyses stratified by high-risk definition type where data permitted, and we explicitly discuss the impact of this heterogeneity on the interpretation of our pooled estimates in the Discussion and Limitations sections. Additionally, we required all included studies to clearly report the timing of D-dimer testing (baseline pre-treatment, during induction therapy, post-treatment remission, or at the onset of thrombotic symptoms), as D-dimer concentrations measured at different clinical time points carry divergent predictive and diagnostic clinical implications. (4) Outcome: D-dimer levels reported as mean and standard deviation, median and interquartile range, or sufficient data to calculate these estimates, allowing computation of standardized mean differences; (5) Study design: Cross-sectional, case-control, or cohort studies that directly compared D-dimer levels between defined MM patient subgroups. Minimum sample size was set at 20 total patients to avoid very small studies with unstable estimates.

Exclusion criteria were: (1) case reports or case series without comparison groups; (2) editorials, conference abstracts without adequate numerical data, reviews, or guideline papers; (3) studies mixing MM with other malignancies without separate MM-specific results; (4) studies measuring D-dimer solely for VTE diagnosis rather than as a biomarker for risk stratification; (5) studies without accessible full text or insufficient numerical data to calculate effect sizes; (6) duplicate publications from the same cohort, in which case the most recent or comprehensive report was retained.

### 2.4 Study selection and data extraction

Two reviewers independently screened titles and abstracts to exclude clearly irrelevant studies. Full texts of potentially relevant articles were then assessed against the predefined eligibility criteria. Disagreements were resolved through discussion or consultation with a third reviewer when necessary. Reasons for exclusion at the full-text stage were documented.

For each included study, we extracted the following data using a standardized form: (1) general information including first author, publication year, country, study design, and sample size; (2) patient characteristics including age, sex distribution, MM type and stage, treatment regimens, and follow-up duration; (3) definition of high-risk and reference groups, with particular attention to whether high-risk was based on ISS stage, VTE occurrence, or mortality prediction; (4) D-dimer measurement details including sample type (plasma versus serum, collection timing), assay methodology (immunoturbidimetric, fluorescent immunoassay, ELISA), and measurement units; this study specifically extracted standardized information on the exact timing of D-dimer blood sampling, categorized as pre-treatment baseline, on-treatment, post-treatment follow-up, or at VTE symptom onset, to distinguish the distinct clinical interpretation of D-dimer values obtained at disparate time windows. (5) D-dimer levels in each group reported as mean and standard deviation or median and interquartile range. When studies reported only medians and ranges, we estimated means and standard deviations using established formulas. Data were extracted independently by two reviewers and cross-checked for accuracy.

### 2.5 Quality assessment

Risk of bias was assessed using domains adapted from the ROBINS-I (Risk Of Bias In Non-randomised Studies of Interventions) tool. Although ROBINS-I was originally designed for non-randomized intervention studies, we adapted it for observational biomarker studies by conceptualizing D-dimer level (or the clinical condition defining the high-risk group) as the “exposure” rather than an intervention. The following domains were evaluated: (1) bias due to confounding and selection of participants, assessing whether group assignment was influenced by factors also related to D-dimer levels; (2) bias in classification of the exposure, evaluating the reliability of high-risk versus reference group assignment based on ISS staging, VTE diagnosis, or mortality ascertainment; (3) bias due to missing data, examining completeness of D-dimer measurements and outcome reporting; (4) bias in measurement of outcomes, evaluating D-dimer assay quality, standardization, and blinding; and (5) bias in selective outcome reporting, assessing whether pre-specified outcomes were fully reported. Each domain was rated as “low risk,” “some concerns,” or “high risk.” Overall risk of bias for each study was judged based on the highest-risk domain. Two reviewers independently performed assessments with disagreements resolved by discussion. Graphical summaries were created using the robvis package in R to generate bar plots and traffic-light plots.

### 2.6 Statistical analysis

To compare D-dimer levels between high-risk and reference groups across studies with different measurement units and scales, we used the standardized mean difference (SMD) as the primary effect measure. SMD values of 0.2, 0.5, and 0.8 are conventionally interpreted as small, medium, and large effect sizes, respectively.

This study performed a random-effects meta-analysis using the inverse-variance method. To account for between-study variability, we used the DerSimonian-Laird estimator with restricted maximum likelihood, and applied the Hartung-Knapp adjustment to provide more conservative confidence intervals given the small number of studies. All pooled estimates are presented with 95% confidence intervals.

To quantify the degree of inconsistency across studies, we calculated the I² statistic and the between-study variance (τ²), along with Cochran’s Q test. I² values of 25%, 50%, and 75% were interpreted as low, moderate, and high heterogeneity, respectively. Prediction intervals were calculated to describe the range of true effects expected in 95% of future similar studies.

Prespecified subgroup analyses were conducted to examine whether results differed by sample type (plasma versus serum) or assay methodology (immunoturbidimetric versus fluorescent immunoassay), using chi-square tests for interaction. To test the robustness of our findings, we performed a leave-one-out sensitivity analysis by sequentially omitting each study and recalculating the pooled estimate.

Publication bias was assessed visually using funnel plots. Formal statistical tests (e.g., Egger’s regression) were not performed due to the small number of studies (k=6), as such tests lack adequate power with fewer than ten studies. A two-sided p-value < 0.05 was considered statistically significant for all analyses. All statistical analyses were performed using R version 4.3.0.

## 3 Results

### 3.1 Study selection

**Figure 2:**
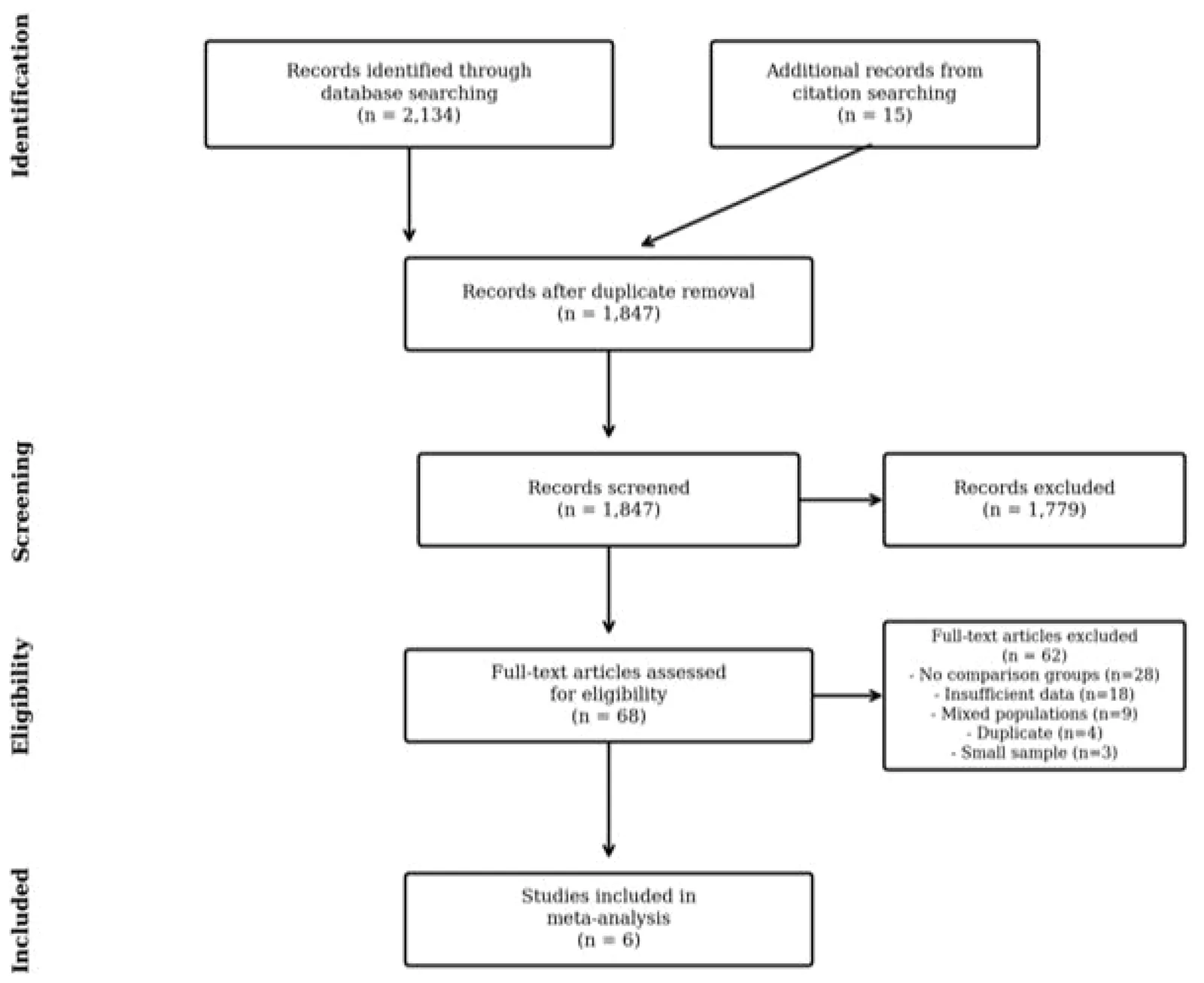
PRISMA flow diagram of study selection.

The systematic search across the four databases identified 2,134 records. An additional 15 records were identified through citation searching of reference lists of relevant reviews and included studies. After removal of 302 duplicates, 1,847 unique records remained for screening. Title and abstract screening excluded 1,779 clearly irrelevant records, leaving 68 articles for full-text review. Of these, 62 were excluded for the following reasons: no comparison between high-risk and reference MM groups (n=28), insufficient quantitative D-dimer data (n=18), mixed with non-MM populations (n=9), duplicate publication (n=4), and sample size below the 20-patient threshold (n=3). Regarding the sample size criterion, three studies with fewer than 20 total participants were identified and excluded, as these were deemed too small to provide stable effect estimates. Ultimately, six studies comprising 697 patients (171 in high-risk groups, 526 in reference groups) met all inclusion criteria and were included in the meta-analysis (18–23). The PRISMA flow diagram detailing the study selection process is presented in **Figure 2**.

### 3.2 Study characteristics

The six included studies were published between 2015 and 2024 and were conducted in China (n=5) and Sudan (n=1) (**Table 1**). All studies employed observational designs including case-control, cross-sectional, and retrospective cohort studies. Sample sizes ranged from 20 to 272 patients. The definition of “high-risk” varied across studies: ISS stage III versus ISS stage I (n=2 studies), VTE occurrence versus no VTE (n=1 study), death versus survival (n=1 study), MM patients versus healthy controls (n=1 study), and abnormal versus normal D-dimer before treatment (n=1 study). It is important to note that the “death versus survival” study used mortality as the outcome being predicted, rather than defining high-risk by a thrombotic event. This distinction is critical, as D-dimer elevation in this context may reflect tumor burden and disease progression rather than acute thrombosis. All studies measured D-dimer in plasma samples. Assay methods included immunoturbidimetric analysis (n=5 studies) and fluorescent immunoassay (n=1 study). Follow-up durations varied from cross-sectional assessments to longitudinal follow-up extending up to 24 months.

**Table 1:** Characteristics of included studies comparing D-dimer levels in high-risk versus reference multiple myeloma patients.

Study	First author	Year	Country	Design	Sample type	Assay method	High-risk definition	N high-risk	N Reference	High-Risk D-dimer	Reference D-dimer
Lin 2015 (18)	Lin	2015	China	Case-control	Plasma	Immunoturbidimetric	MM patients	68	199	Higher	Lower (healthy controls)
Zhang 2018 (19)	Zhang	2018	China	Cross-sectional	Plasma	Immunoturbidimetric	ISS III	17	10	Elevated	Lower (ISS I)
Hussein 2020 (20)	Hussein	2017	Sudan	Cross-sectional	Plasma	Fluorescent immunoassay	Abnormal D-dimer pre-treatment	12	18	Abnormal	Normal
Ji 2021 (21)	Ji	2021	China	Retrospective cohort	Plasma	Immunoturbidimetric	VTE occurrence	51	221	Elevated in VTE group	Lower in non-VTE
Chen 2024 (22)	Chen	2024	China	Cross-sectional	Plasma	Immunoturbidimetric	ISS III	11	20	Elevated	Lower (ISS I)
Zhu 2023 (23)	Zhu	2023	China	Retrospective cohort	Plasma	Immunoturbidimetric	Death	12	58	Elevated in death group	Lower in survivors

ISS, International Staging System; MM, multiple myeloma; SD, standard deviation; VTE, venous thromboembolism.

### 3.3 Risk of bias assessment

**Figure 3:**
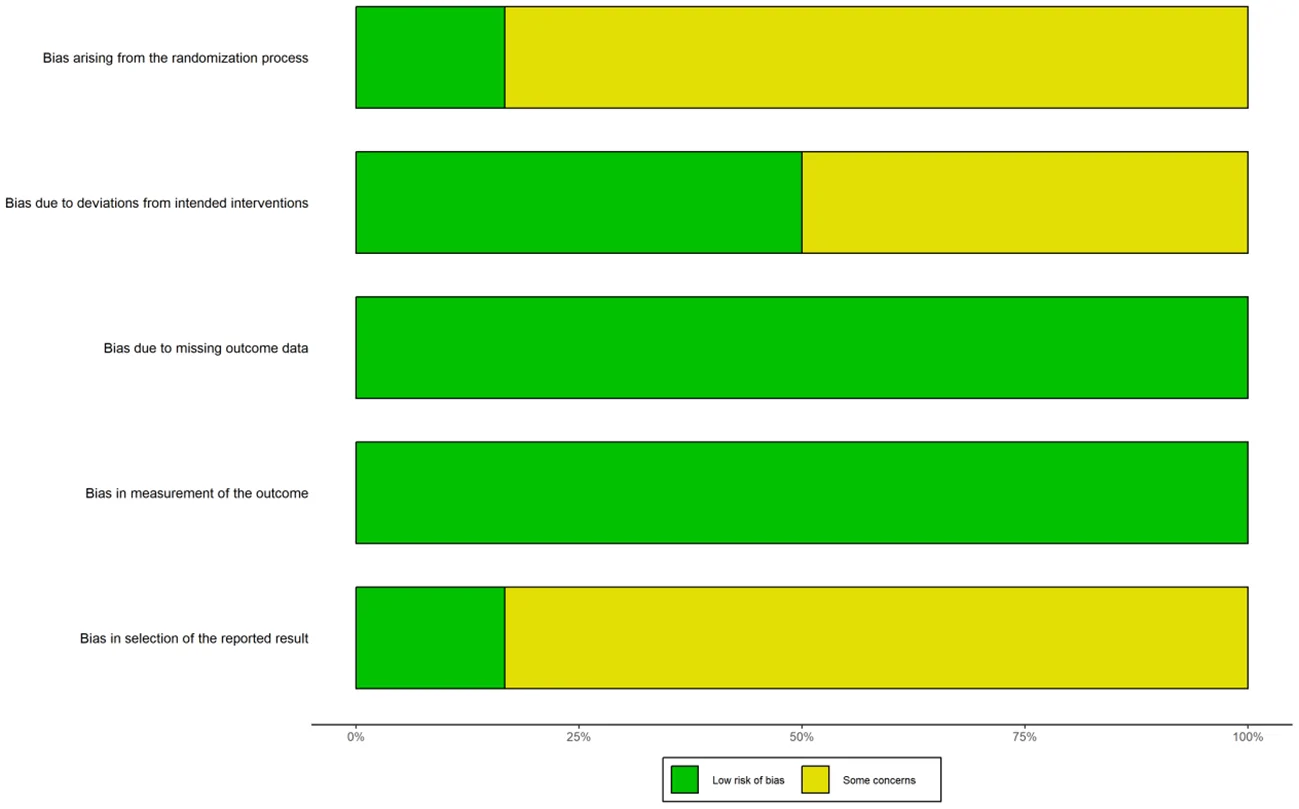
Risk of bias summary: domain-level judgments across studies.

**Figure 4:**
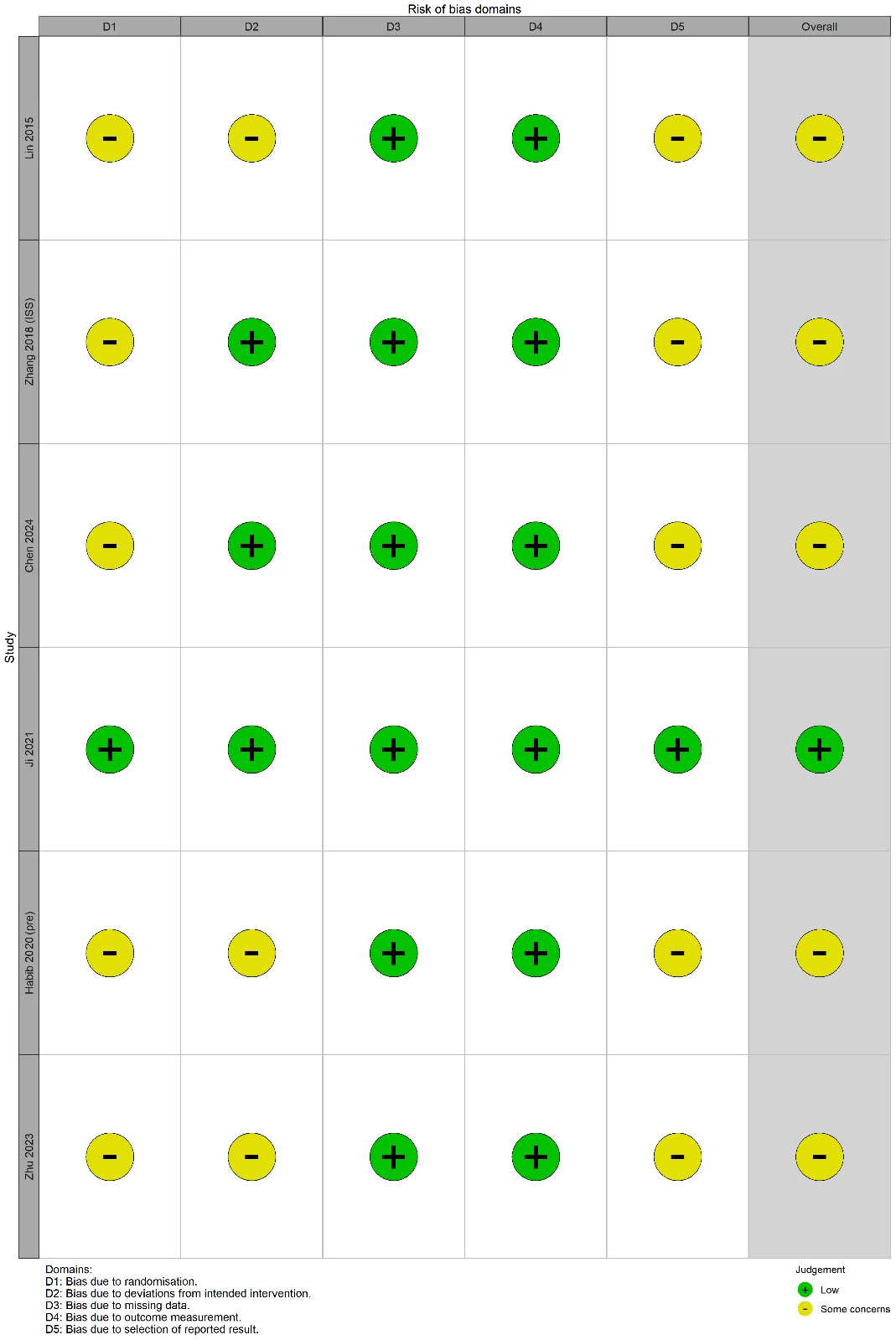
Risk of bias assessment: traffic light plot.

Risk of bias assessment revealed methodological limitations across included studies (**Figures 3**, **4**). Most studies showed “some concerns” or “high risk” in domains related to confounding and participant selection, reflecting their observational nature and potential for selection bias. Classification of exposure (group assignment) generally received “low risk” or “some concerns” ratings, as ISS staging and VTE diagnosis followed established criteria. Outcome measurement domains generally showed “low risk,” as D-dimer assays are objective and standardized. Missing data concerns were minimal. Overall, the predominance of “some concerns” ratings underscores the need for cautious interpretation of findings and highlights the necessity for prospective, well-designed studies.

### 3.4 Primary outcome: D-dimer levels in high-risk versus reference groups

**Figure 5:**
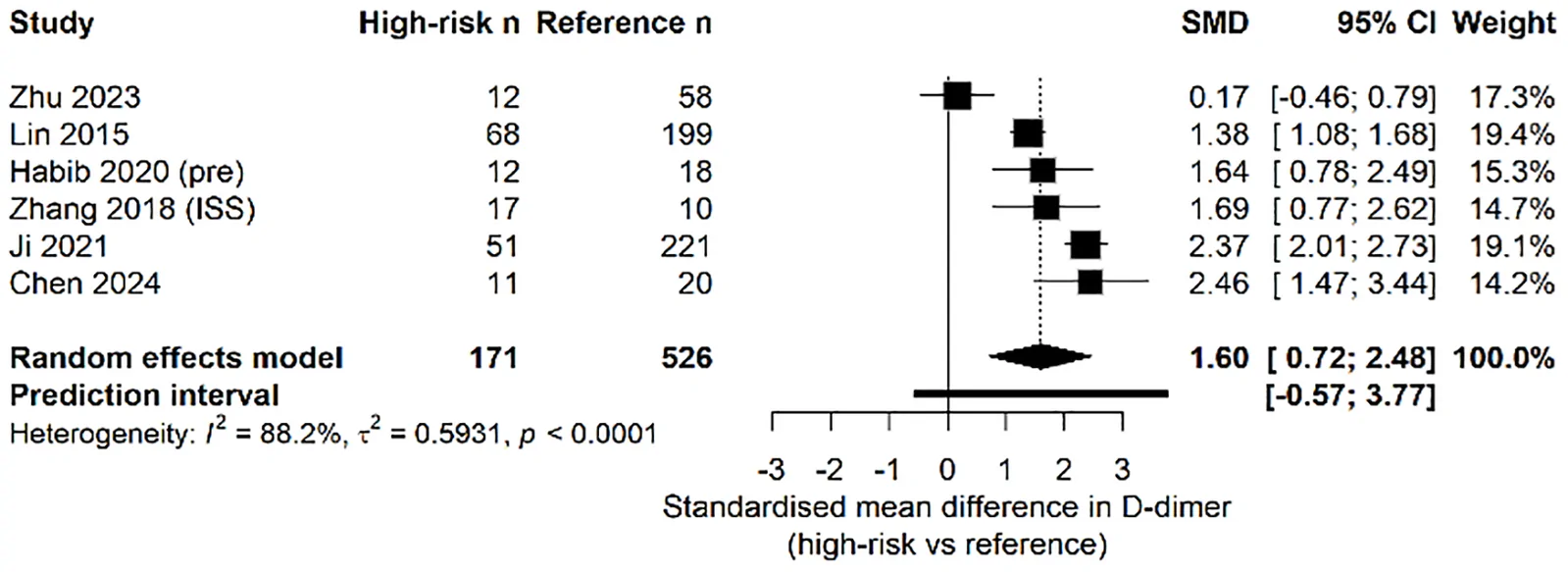
Forest plot of standardized mean difference in D-dimer levels: high-risk versus reference multiple myeloma patients.

The primary meta-analysis pooled SMD estimates from all six studies (**Figure 5**). Across all six studies, high-risk MM patients consistently showed substantially higher D-dimer levels than reference groups. The magnitude of this difference was large (SMD 1.60, 95% CI 0.72–2.48, p<0.0001). The common-effect model yielded similar results (SMD 1.28, 95% CI 1.18–1.39). However, the results varied considerably across studies (I²=88.2%, p<0.0001), which is not unexpected given that the included studies categorized “high-risk” using biologically and clinically distinct definitions: ISS stage III (reflecting tumor burden-driven chronic hypercoagulability), VTE occurrence (reflecting acute thrombotic events), and mortality (reflecting overall disease aggression). These divergent definitions introduce substantial clinical and biological heterogeneity, which is reflected in the high I² statistic. The prediction interval ranged from −0.57 to 3.77, indicating that while most future studies would likely show elevated D-dimer in high-risk groups, some variability is expected depending on which high-risk phenotype is being studied.

### 3.5 Subgroup analyses

**Figure 6:**
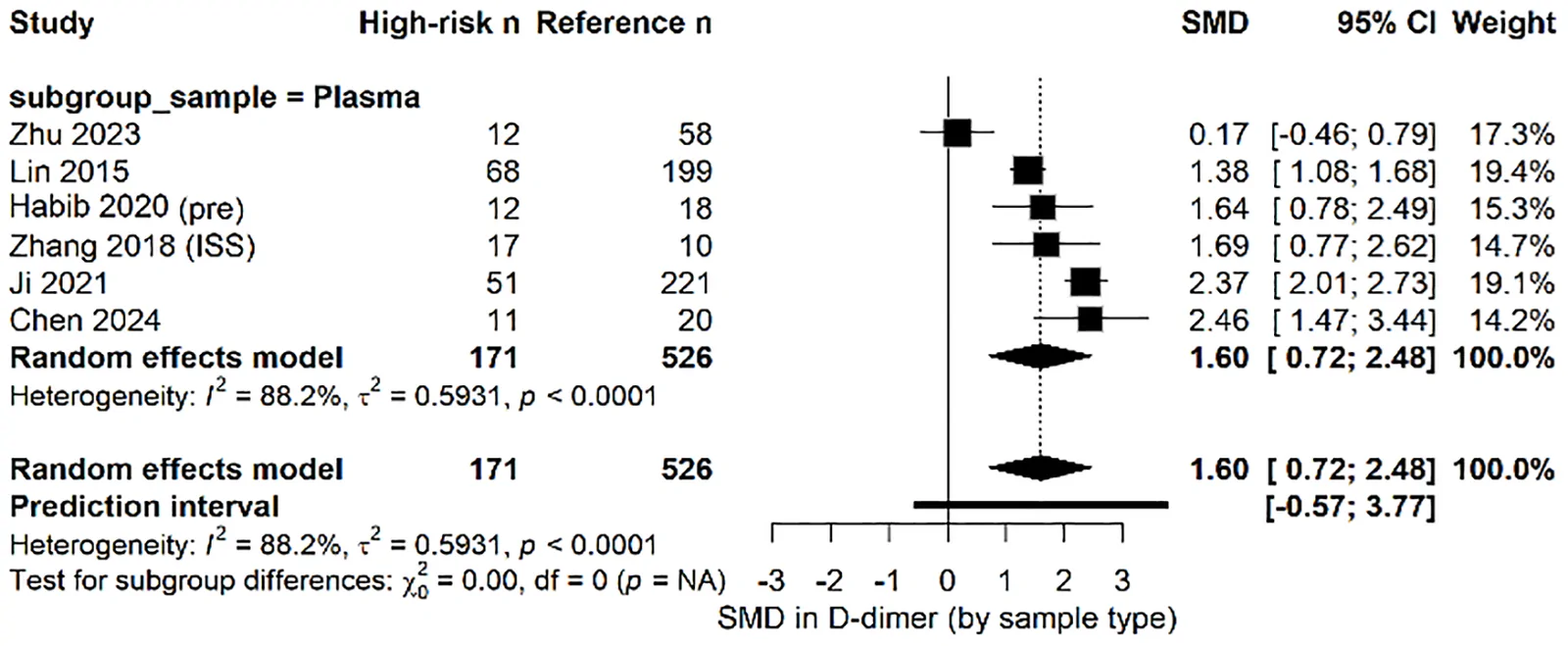
Subgroup analysis by sample type.

Subgroup analysis by sample type: All six included studies used plasma samples, precluding meaningful subgroup comparison (**Figure 6**). The pooled SMD for plasma samples was identical to the overall analysis (SMD 1.60, 95% CI 0.72–2.48, I²=88.2%). This uniformity in sample type eliminates pre-analytical variability as a source of heterogeneity.

**Figure 7:**
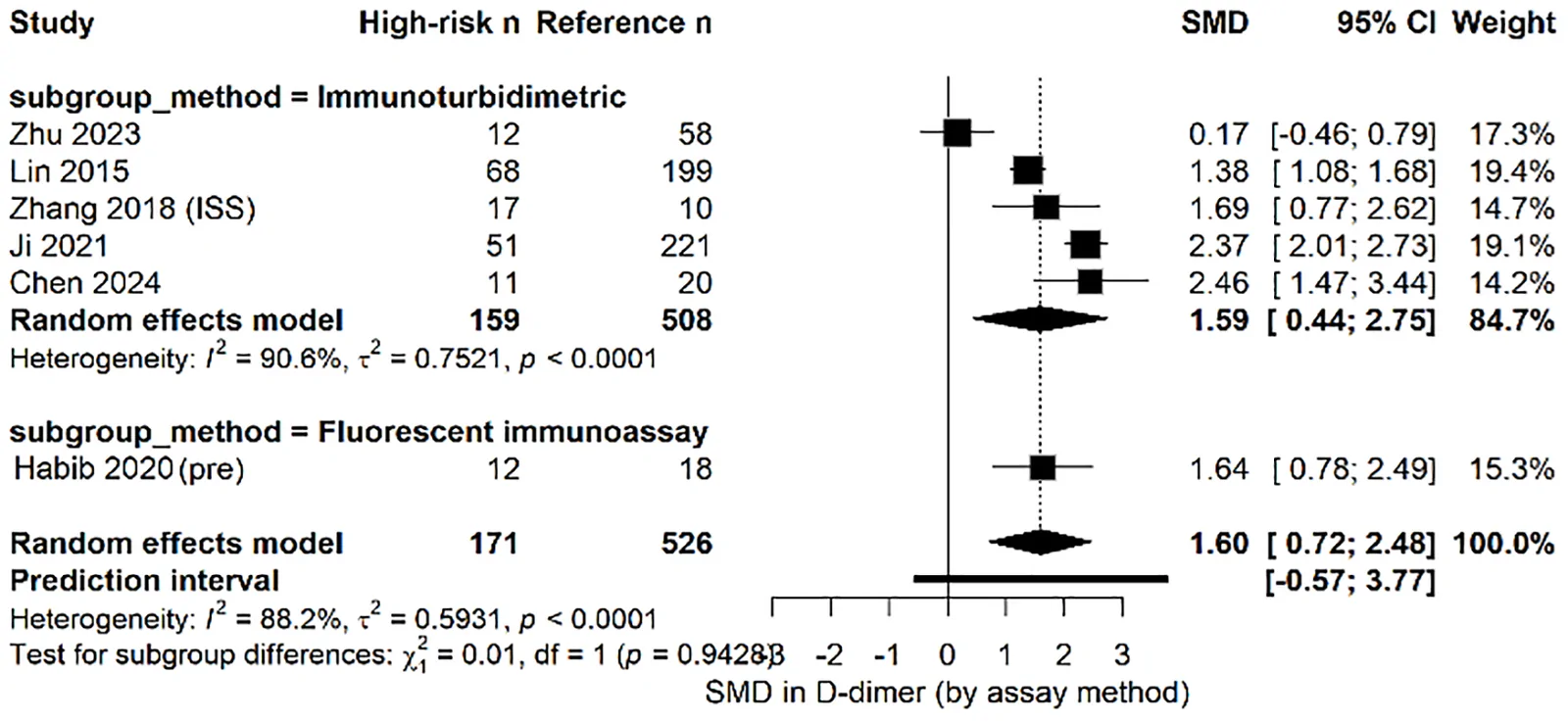
Subgroup analysis by assay methodology.

Subgroup analysis by assay method: Five studies employed immunoturbidimetric methods (SMD 1.59, 95% CI 0.44–2.75, I²=90.6%), while the single study using fluorescent immunoassay showed a similarly large effect (SMD 1.64, 95% CI 0.78–2.49) (**Figure 7**). Both methods demonstrated large effect sizes favoring elevated D-dimer in high-risk patients, with overlapping confidence intervals suggesting no significant method-related differences (test for subgroup differences: χ²=0.01, df=1, p=0.943). The consistency across assay platforms supports the robustness of findings and suggests that D-dimer elevation in high-risk MM is detectable regardless of analytical method.

### 3.6 Sensitivity analysis

**Figure 8:**
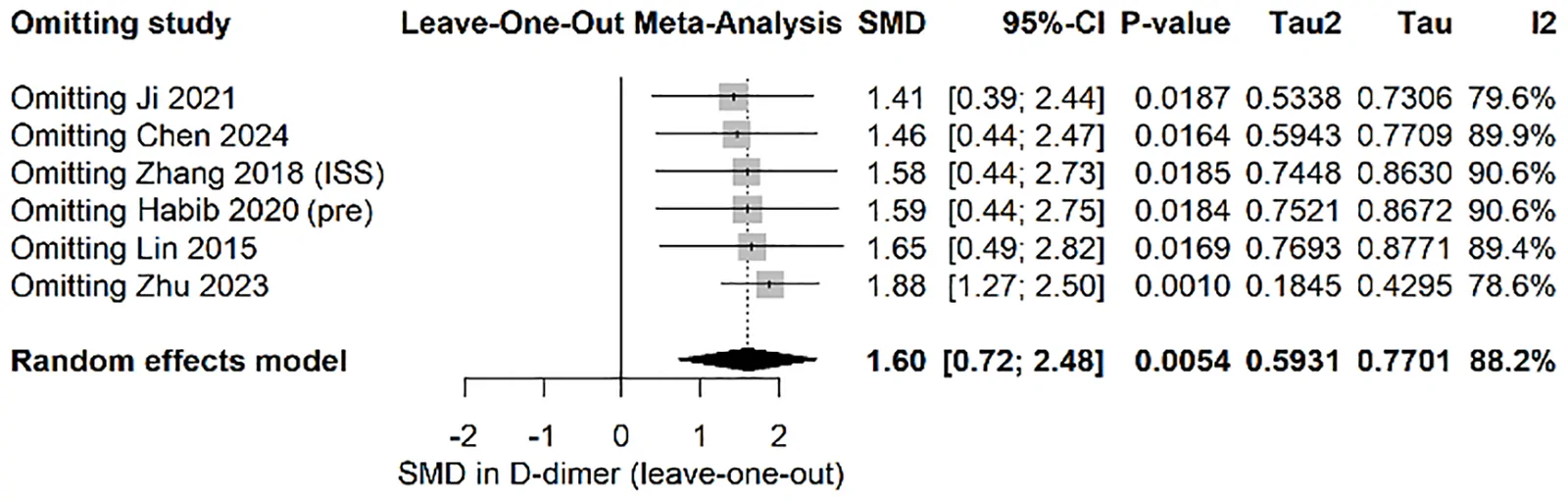
Leave-one-out sensitivity analysis.

To test whether any single study drove the overall result, we sequentially removed each study and recalculated the pooled effect. This analysis confirmed that no individual study disproportionately influenced the findings (**Figure 8**). Sequential omission of each study yielded pooled SMDs ranging from 1.41 to 1.88, with all confidence intervals excluding zero and maintaining statistical significance (all p<0.05). The corresponding I² values ranged from 78.6% to 90.6%, indicating that while some heterogeneity persisted, the overall conclusion remained unchanged. This robustness analysis confirms that the observed association between high-risk MM status and elevated D-dimer is not driven by any individual study and represents a consistent pattern across the literature.

### 3.7 Publication bias assessment

A visual inspection of the funnel plot revealed a reasonably symmetric distribution of studies around the pooled estimate, with no obvious asymmetry suggesting publication bias or small-study effects (**Figure 1**). Formal statistical tests for funnel plot asymmetry were not performed due to the limited number of studies (k=6), as such tests lack adequate power with fewer than ten studies and may yield misleading results. Therefore, the visual assessment should be interpreted with appropriate caution, and we cannot definitively exclude the presence of publication bias. Nevertheless, the absence of gross asymmetry provides limited reassurance against major systematic bias in the evidence base.

## 4 Discussion

This systematic review and meta-analysis demonstrates that high-risk MM patients exhibit substantially elevated D-dimer levels compared to lower-risk populations, with a pooled standardized mean difference of 1.60 (95% CI 0.72–2.48), representing a large effect size. This finding remained robust across sensitivity analyses, with consistent elevation observed regardless of assay methodology. The magnitude of this difference suggests that D-dimer may correlate with clinical features that identify MM patients at increased risk for adverse outcomes including thrombotic events, disease progression, and mortality, rather than definitively causally predict these poor endpoints. Although we have established statistically significant D-dimer disparities between high-risk and low-risk MM cohorts, the translational value of these statistical results for routine clinical practice requires comprehensive elaboration. We herein detail two core clinical applications of our findings: first, how D-dimer complements and upgrades conventional prognostic frameworks (ISS, R-ISS) to refine long-term risk stratification; second, how D-dimer modifies IMPEDE-VTE guided thromboprophylaxis tactics to deliver individualized anticoagulation plans for myeloma patients.

The observed large effect size (SMD >1.5) deserves emphasis, as it substantially exceeds conventional thresholds for clinical significance. In comparison, many established cancer biomarkers demonstrate small-to-medium effect sizes (SMD 0.2-0.5) when comparing prognostically distinct patient subgroups (24). The magnitude of D-dimer elevation in high-risk MM patients approaches that observed for highly discriminatory tumor markers and suggests genuine biological differences in coagulation activation and fibrinolytic activity. This is consistent with the pathophysiology of advanced MM, where higher tumor burden, greater bone marrow infiltration, increased inflammatory cytokine production, and more intensive treatment regimens collectively drive a prothrombotic state (25).

The consistency of findings across different high-risk definitions (ISS III staging, VTE occurrence, mortality) is noteworthy but warrants nuanced interpretation. The divergent definitions used to categorize “high-risk” MM across included studies represent a major source of the substantial between-study statistical heterogeneity identified in this meta-analysis (I²=88.2%), and each definition corresponds to biologically distinct clinical endpoints linked to separate mechanisms of D-dimer elevation. Given the extremely high I² value (88.2%), a more comprehensive dissection of potential heterogeneity contributors beyond inter-assay differences is required. Apart from analytical platform variability, multiple key clinical and methodological confounders jointly drive the prominent between-study inconsistency, including heterogeneous baseline patient demographic and comorbidity profiles, divergent anti-myeloma treatment regimens adopted across cohorts, uneven distribution of disease stages within composite high-risk groups, and inconsistent timing of D-dimer blood collection relative to diagnosis, treatment cycles or thrombotic symptom onset. This heterogeneity is not a statistical artifact but rather reflects genuine biological and clinical diversity: the elevated D-dimer observed in ISS stage III patients primarily reflects chronic tumor burden-driven hypercoagulability, whereas the elevation seen in patients with VTE represents an acute thrombotic and fibrinolytic response, and the elevation observed in non-survivors may reflect a combination of both processes along with systemic illness severity. Pooling these biologically distinct subgroups under a single “high-risk” umbrella was a necessary compromise given the limited available literature, but it directly explains the substantial I² value and limits the precision of our pooled estimate for any specific clinical application. In patients with acute VTE, D-dimer elevation primarily reflects direct activation of the coagulation cascade and subsequent fibrinolysis, representing an acute thrombotic process. In contrast, ISS stage III patients demonstrate elevated D-dimer more likely driven by chronic mechanisms including high tumor burden with associated tissue factor expression by malignant plasma cells, endothelial dysfunction from paraprotein deposition, and systemic inflammatory cytokine release. Patients who died may have had elevated D-dimer reflecting both acute thrombotic complications and chronic disease-related hypercoagulability. Pooling these heterogeneous patient subgroups under a single “high-risk” composite category was a necessary methodological compromise given the limited volume of published literature, yet this grouping masks meaningful differences in the magnitude and biological drivers of D-dimer elevation across distinct clinical risk phenotypes and directly contributes to the extreme statistical heterogeneity detected. Consequently, the pooled SMD of 1.60 should be interpreted as a general signal that D-dimer is elevated across various high-risk MM phenotypes, rather than as a precise quantitative estimate applicable to any single high-risk definition. From the perspective of optimizing ISS and R-ISS staging systems: neither classic ISS nor revised R-ISS incorporates markers of hypercoagulability, even though thrombosis independently worsens myeloma survival. Our pooled data confirms ISS III patients carry markedly higher D-dimer than ISS I counterparts, which enables auxiliary risk stratification within identical staging tiers. For two patients assigned the same R-ISS grade, a significantly elevated baseline D-dimer can flag a subpopulation with compounded risk of progression and death, requiring intensified monitoring intervals and more frequent reassessment of treatment response. Additionally, serial D-dimer testing can dynamically track disease activity during treatment, filling the static limitation of one-time ISS/R-ISS baseline staging. ISS stage III patients, characterized by high beta-2 microglobulin levels indicating tumor burden and renal impairment, showed markedly elevated D-dimer compared to ISS stage I patients, suggesting a direct relationship between disease severity and coagulation activation. Similarly, MM patients who developed VTE had higher baseline D-dimer than those who remained VTE-free, validating previous reports that D-dimer predicts thrombotic events in this population (26). The elevation in patients who died compared to survivors further implicates D-dimer as a marker of overall disease aggression and poor prognosis, consistent with findings in other malignancies where hypercoagulability correlates with mortality independent of thrombotic events (27). For IMPEDE-VTE-based thromboprophylaxis management: the original scoring model only relies on static clinical variables without real-time coagulation readouts. Our statistically robust intergroup difference supports integrating D-dimer as a dynamic risk modifier to adjust anticoagulation strategies: (1) Patients with low IMPEDE-VTE score but elevated D-dimer should switch anti-thrombotic prophylaxis from aspirin to low-molecular-weight heparin; (2) Patients with intermediate/high IMPEDE-VTE risk plus raised D-dimer require prolonged LMWH coverage extending past induction therapy; (3) In cases of suspected acute VTE, elevated D-dimer takes priority over baseline IMPEDE-VTE risk tier to trigger urgent radiological evaluation.

It is critical to distinguish between D-dimer’s role in predicting VTE versus its role as a marker of tumor burden and treatment response. Previous studies have established that baseline pre-treatment D-dimer levels can predict incident VTE in MM patients, supporting its utility in upfront thromboprophylaxis decision-making. Conversely, serial D-dimer monitoring during active treatment may dynamically reflect tumor response, as effective therapy reduces tumor burden and associated procoagulant stimuli; a recent multicenter retrospective cohort study validated that longitudinal plasma D-dimer changes could reliably evaluate treatment response in MM patients (28). D-dimer measured at the emergence of limb or respiratory symptoms acts as an acute diagnostic marker for suspected VTE, with interpretative thresholds distinct from steady-state baseline values. This meta-analysis, which pools studies using different high-risk definitions, cannot disentangle these distinct roles. However, the uniformly elevated D-dimer observed across all high-risk subgroups suggests that D-dimer is a sensitive but non-specific biomarker that is elevated in both acute thrombotic states and chronic disease burden states. Future prospective studies should separately evaluate D-dimer’s performance for VTE prediction versus treatment response monitoring, as these represent distinct clinical applications requiring different thresholds and interpretative frameworks based on standardized sampling timing.

The substantial heterogeneity observed (I²=88.2%) warrants careful consideration. While statistical heterogeneity was high, the direction of effect was uniformly positive across all studies, with confidence intervals for individual studies mostly overlapping. As noted above, the primary driver of this heterogeneity is the clinical and biological diversity of the “high-risk” definitions used across the included studies, rather than methodological or analytical differences. Beyond assay-related variance, four critical confounding dimensions jointly fuel the extreme between-study inconsistency: 1) Patient characteristic variability: Studies enrolled participants with divergent age distributions, baseline renal function, and comorbidity burdens; chronic kidney disease and advanced age independently elevate baseline D-dimer and create inter-cohort imbalance; 2) Treatment plan heterogeneity: Immunomodulatory agents carry far stronger prothrombotic effects than proteasome inhibitors alone, yet included studies adopted inconsistent induction/maintenance regimens without uniform subgroup stratification; 3) Uneven disease stage composition within pooled high-risk arms: Some high-risk cohorts predominantly contained ISS III patients, while others focused on post-VTE or fatal cases, generating divergent average D-dimer magnitudes; 4) Non-standardized D-dimer measurement timing: Specimens collected pre-diagnosis, pre-induction, mid-treatment or at acute thrombus onset yield drastically different D-dimer concentrations, yet few studies harmonized sampling windows. This is supported by our subgroup analysis by assay methodology, which showed no significant differences between immunoturbidimetric and fluorescent immunoassay methods, suggesting that heterogeneity does not stem from analytical platform differences. Consistent D-dimer elevation across all mainstream laboratory assays removes technical barriers to universal clinical implementation, allowing hospitals to combine D-dimer testing with routine ISS/R-ISS lab panels and IMPEDE-VTE risk assessment at initial myeloma diagnosis. Sources of heterogeneity likely include differences in high-risk group definitions (ISS III versus VTE versus mortality), treatment regimens which impact thrombotic risk differentially (particularly immunomodulatory drug exposure), patient characteristics including age, comorbidities, and performance status, the timing of D-dimer measurement relative to diagnosis, treatment initiation, or venous thromboembolism symptom onset—an important confounding factor ignored in most prior pooled analyses, as baseline pre-treatment D-dimer reflects steady-state tumor-related hypercoagulability, on-treatment D-dimer fluctuates with drug-induced prothrombotic effects, and D-dimer tested at acute VTE onset primarily signals acute fibrinolytic activation, and laboratory-specific reference ranges and measurement variability. Future studies should aim to stratify analyses by specific high-risk definitions to provide clinically meaningful estimates for each distinct patient subgroup. Importantly, subgroup analysis by assay methodology revealed no significant differences, suggesting that heterogeneity does not stem from analytical platform differences but rather from genuine clinical and biological variability across study populations. If future studies permit, subgroup analyses stratified by specific high-risk definitions (e.g., ISS III vs. ISS I only, or VTE vs. no VTE only) and standardized D-dimer sampling timing would help clarify in which clinical time windows D-dimer demonstrates the strongest discriminatory value for risk stratification.

Our findings carry tentative, hypothesis-generating clinical implications that require prospective validation before routine clinical adoption, given the limited total number of included studies (n=6) and the exclusively observational, non-interventional design of all available evidence. First, they provide empirical support for incorporating D-dimer measurement into risk stratification algorithms for MM patients. The IMPEDE VTE score and its D-dimer-enhanced derivative (IMPEDED VTE) have demonstrated improved prediction of thrombotic events (29), and our meta-analysis validates the biological rationale underlying these models by confirming that high-risk patients have markedly elevated D-dimer. Second, the large effect size suggests that D-dimer cutoffs could potentially be developed to distinguish patients with adverse clinical profiles, though the heterogeneity of high-risk definitions across cohorts means no universal threshold can be reliably proposed from the current dataset for clinical use. Third, serial D-dimer monitoring might theoretically support dynamic risk reassessment as patients transition through treatment phases, yet longitudinal prospective data to confirm this utility remains absent from the existing literature.

The results also raise important questions about underlying mechanisms. Elevated D-dimer in MM may reflect direct procoagulant activity of malignant plasma cells expressing tissue factor and cancer procoagulant, inflammatory cytokine-mediated endothelial activation and tissue factor expression, impaired fibrinolytic capacity due to elevated plasminogen activator inhibitor-1, treatment-induced hypercoagulability particularly from immunomodulatory drugs, hyperviscosity from high paraprotein levels causing blood stasis, and immobility from skeletal complications. It remains unclear whether D-dimer elevation mediates adverse clinical outcomes or solely acts as a passive correlative biomarker of underlying disease severity; no causal inference can be drawn from the observational data synthesized here. Prospective studies with comprehensive hemostatic panels could disentangle these mechanisms and identify which patients might benefit from specific interventions targeting hypercoagulability (30).

### 4.1 Limitations

Several important limitations of this meta-analysis merit acknowledgment. First, the small number of included studies (k=6) limits statistical power for subgroup analyses and publication bias assessment. The extreme between-study heterogeneity (I²=88.2%) further restricts the reliability of stratified analyses to isolate the independent impact of patient profiles, treatment modalities, disease stage distribution and sampling timing, as the limited sample of studies cannot support fully adjusted multivariable meta-regression to quantify each heterogeneity source individually. With only six studies, formal tests such as Egger’s regression lack adequate power to detect funnel plot asymmetry reliably, and the visual assessment of the funnel plot should be interpreted cautiously. While our search was comprehensive, the field may suffer from reporting bias whereby negative or null findings remain unpublished. Second, all included studies were observational with inherent risks of confounding, selection bias, and residual confounding from unmeasured variables. The predominance of retrospective designs and “some concerns” or “high risk” ratings in bias assessment underscore these limitations, meaning all observed associations between D-dimer and high-risk MM cannot be interpreted as causal relationships. Third, the most impactful source of clinical heterogeneity stems from inconsistent, non-standardized definitions of “high-risk” MM across individual investigations: studies categorized high-risk patients using three distinct clinical endpoints (advanced ISS stage, incident VTE, all-cause mortality), each driven by separate thrombotic and tumor-burden related pathophysiological pathways. This lack of uniform risk stratification criteria across primary studies amplifies statistical heterogeneity and prevents reliable quantification of D-dimer effect sizes for individual adverse clinical outcomes in isolation. Consequently, the pooled SMD of 1.60 represents an average effect across heterogeneous high-risk phenotypes, which may not accurately reflect the true effect size for any specific high-risk definition. For example, the D-dimer elevation in patients with acute VTE may be substantially larger than in patients with ISS stage III disease without thrombosis, and our pooled estimate obscures these clinically important differences. D-dimer may be very high in patients with acute thrombosis but only modestly elevated in patients with advanced-stage disease, and pooling these distinct groups limits the precision of our estimates. As a result, our findings should be considered hypothesis-generating, and the pooled estimate should not be used to derive a universal D-dimer cutoff for clinical decision-making across all high-risk MM subgroups. Fourth, we could not perform stratified analyses by specific treatment regimens (immunomodulatory drugs versus proteasome inhibitors versus combination therapies) or by standardized D-dimer measurement timing (baseline pre-diagnosis, pre-treatment, on-treatment, or acute VTE presentation), two key clinical variables that exert profound impacts on D-dimer concentration and its corresponding clinical meaning. Without uniform reporting of sampling timepoints, we were unable to quantify whether the prognostic utility of D-dimer differs when measured at disease baseline versus during active treatment or at thrombotic event onset. Fifth, the review protocol was not prospectively registered in PROSPERO, which may reduce transparency and increase the potential for outcome reporting bias, although all analyses were prespecified. Sixth, all studies originated from China, Sudan, or Egypt, potentially limiting generalizability to other populations with different genetic backgrounds, healthcare systems, laboratory practices, and treatment patterns. The absence of data from Western, East Asian (non-Chinese), or Latin American populations represents an important gap, and future multicenter international studies are needed to confirm whether these findings are generalizable across diverse ethnic and clinical settings (31).

Future research should address these gaps through several approaches. To resolve the extreme heterogeneity observed in the current pooled analysis, subsequent large-scale meta-analyses and primary cohort studies must standardize four key dimensions: consistent high-risk categorization criteria, unified D-dimer specimen collection timing, documented uniform anti-myeloma treatment stratification, and balanced adjustment for patient baseline comorbidities and age. Large, prospective cohort studies with standardized D-dimer measurement at pre-specified, clearly documented timepoints (diagnosis baseline, prior to each treatment cycle, post-treatment restaging, and upon suspicion of VTE) would minimize bias and allow adjustment for comprehensive confounders including treatment regimens, performance status, and comorbidities. Establishing validated D-dimer cutoffs specific to MM populations, possibly stratified by ISS stage and treatment regimen and testing timing, would enable clinical decision-making regarding thromboprophylaxis intensity. Comparative effectiveness research evaluating D-dimer-guided versus standard thromboprophylaxis strategies could assess whether biomarker-directed approaches improve outcomes, with separate cutoffs validated for baseline risk prediction and acute VTE suspicion scenarios. Integration of D-dimer with other emerging biomarkers including tissue factor-bearing microvesicles, procoagulant phospholipids, and genetic thrombophilia markers might yield multivariable models with superior discrimination. Finally, mechanistic studies examining relationships between D-dimer levels, specific MM cytogenetic subtypes, and hemostatic pathway activation would advance biological understanding and potentially identify therapeutic targets.

## 5 Conclusions

This meta-analysis of six studies comprising 697 patients demonstrates that MM patients with poor prognostic features (e.g., ISS stage III) or who experience VTE exhibit substantially elevated D-dimer levels compared to lower-risk reference groups (SMD 1.60, 95% CI 0.72–2.48, p<0.0001). This large and robust effect size supports D-dimer’s potential as a clinically relevant biomarker for disease severity and thrombotic risk stratification in MM. Taken together, our statistically significant intergroup differences deliver actionable updates to daily myeloma clinical practice: D-dimer acts as an auxiliary marker to refine risk stratification within fixed ISS/R-ISS tiers, and serves as a dynamic modifier to adjust anticoagulation intensity and duration when incorporated into IMPEDE-VTE risk assessment, thereby personalizing routine thromboprophylaxis strategies for individual patients. Notably, the extremely high heterogeneity (I²=88.2%) observed in our pooled analysis cannot be explained by assay differences alone; substantial confounding originates from inconsistent patient baseline characteristics, variable treatment schedules, uneven disease stage composition and non-standardized D-dimer sampling timing across primary studies, which must be controlled in future research to generate more precise, phenotype-specific effect estimates. However, the heterogeneous definitions of “high-risk” across studies-encompassing tumor burden, thrombotic events, and mortality—together with inconsistent reporting of D-dimer sampling timing across primary trials, preclude a unified interpretation. D-dimer elevation measured at acute VTE onset reflects a distinct acute pathophysiological process compared to steady-state elevation driven by high tumor burden in advanced-stage disease captured via pre-treatment baseline testing. Future studies should clearly distinguish between D-dimer’s role in predicting thrombotic events versus monitoring tumor burden and treatment response, and must explicitly report the exact timing of D-dimer specimen collection to allow stratified analyses aligned with each biomarker’s unique clinical purpose. This consistent correlative signal across observational cohorts provides preliminary proof-of-concept that D-dimer merits further evaluation as a biomarker correlated with disease severity and thrombotic risk stratification in MM, yet definitive clinical implementation cannot be recommended based solely on the limited, heterogeneous observational evidence currently available. Future prospective studies should establish standardized D-dimer cutoffs and evaluate D-dimer-guided management strategies in diverse populations.

## Data Availability

The raw data supporting the conclusions of this article will be made available by the authors, without undue reservation.
